# Transcriptome analysis revealed enrichment pathways and regulation of gene expression associated with somatic embryogenesis in *Camellia sinensis*

**DOI:** 10.1038/s41598-023-43355-9

**Published:** 2023-09-24

**Authors:** Hao-Zhen Li, Hui Wu, Kang-Kang Song, Hui-Hui Zhao, Xiao-Yan Tang, Xiao-Hua Zhang, Di Wang, Shao-Lin Dong, Feng Liu, Jun Wang, Zhong-Cong Li, Long Yang, Qin-Zeng Xiang

**Affiliations:** 1 College of Plant Protection and Agricultural Big-Data Research Center, Shandong Agricultural University, Tai’an, 271018 China; 2 College of Horticulture Science and Engineering, Shandong Agricultural University, Tai’an, 271018 China; 3 AgricultureIsLife, Gembloux Agro-Bio Tech, Liege University, 5030 Gembloux 2, Belgium; 4 Ri Zhao Cha Cang Tea Co. Ltd, Ri’zhao, 276800 China; 5 State Key Laboratory of Tea Plant Biology and Utilization, Anhui Agricultural University, He’fei, 230036 China

**Keywords:** Plant sciences, Plant reproduction

## Abstract

The high frequency, stable somatic embryo system of tea has still not been established due to the limitations of its own characteristics and therefore severely restricts the genetic research and breeding process of tea plants. In this study, the transcriptome was used to illustrate the mechanisms of gene expression regulation in the somatic embryogenesis of tea plants. The number of DEGs for the (IS intermediate stage)_PS (preliminary stage), ES (embryoid stage)_IS and ES_PS stages were 109, 2848 and 1697, respectively. The enrichment analysis showed that carbohydrate metabolic processes were considerably enriched at the ES_IS stage and performed a key role in somatic embryogenesis, while enhanced light capture in photosystem I could provide the material basis for carbohydrates. The pathway analysis showed that the enriched pathways in IS_PS process were far less than those in ES_IS or ES_PS, and the photosynthesis and photosynthetic antenna protein pathway of DEGs in ES_IS or ES_PS stage were notably enriched and up-regulated. The key photosynthesis and photosynthesis antenna protein pathways and the Lhcb1 gene were discovered in tea plants somatic embryogenesis. These results were of great significance to clarify the mechanism of somatic embryogenesis and the breeding research of tea plants.

## Introduction

Somatic embryogenesis is a mode of stimulating plant cell totipotency^1^ and is considered to be the most efficient morphogenetic pathway for plant reproduction^2^. Gymnosperm embryogenesis, a significant breakthrough in plant tissue culture during the late twentieth century^3,4^, offers numerous advantages over organ differentiation as a method of plant regeneration. These advantages include a high number of embryos, rapid development, structural integrity, and a high rate of regeneration^5–7^. As a result, gymnosperm embryogenesis has found extensive applications in various fields, serving as a reliable and efficient plant regeneration system and an ideal recipient system for genetic transformation^8,9^.

The molecular biology and bioinformatics aspects of plant somatic embryogenesis has been extensively explored in order to elucidate the mechanisms involved. The formation of somatic embryos in sweet pepper (*Capsicum annuum*) was stimulated with the developmental regulator *PLETHORA* (*PLT5*) by in vitro tissue culture^10^. In the model plant *Arabidopsis thaliana*, the somatic embryo had been studied to originate from the leaf primordium next to the apical meristem of the stem, and growth hormone treatment could efficiently induce embryogenic responses in explants cultured in vitro^11,12^. Transcriptome analysis was used to address the low reproductive rate of peony (*Peaonia ostii*) and revealed that genes determining cell fate and cell division dominated the formation of somatic embryos in peony^13^. Transcriptomic data from maize (*Zea mays* L.) embryonic healing tissue and somatic embryos were studied to reveal hormonal signalling pathways and transcriptional regulation associated with somatic embryogenesis^14^.

*Camellia sinensis* (L.) O. Kuntze is a perennial evergreen plant belonging to the Camelliaceae family^15^. It has been cultivated for centuries and its tea leaves are highly prized for their economic value. Tea plants possess characteristic secondary metabolites such as tea polyphenols^16^, catechins and caffeine^17^, which have many health benefits^18^, such as cancer prevention and treatment of cardiovascular diseases^19^. Tea plants are self-incompatible and unaffiliated, which lead to their high degree of heterozygosity^20,21^. Traditional breeding methods for tea have the disadvantages of being labour-intensive, blind and long breeding cycles^22,23^, while the introduction of modern molecular techniques can speed up the selection process of good tea varieties^24–27^. The establishment of a high-frequency somatic embryogenesis system for tea is of significant practical importance. It allows for the in vitro conservation of excellent tea germplasm resources and the development of genetic transformation and transgenic systems for tea plants.

Due to the polyphenol-rich nature of tea plants and the characteristics of perennial woody plants^16^, the tissue culture process of tea plants suffered from severe browning and poor reproducibility, and the difficulties in obtaining regenerated intact plants^28–30^. Therefore, the high frequency and stable somatic embryogenesis system of tea plants has not been established so far, which greatly affects and limits the development of cell engineering and genetic engineering of tea plants and other related researches. At present, more studies on somatic embryogenesis in horticultural plants have been conducted, and the technical methods are more mature^31–33^, but the researches on somatic embryogenesis in tea were less systematic. The majority of current studies have focused on determining the optimal conditions for somatic embryogenesis, including the ratio of plant growth regulators and the combination of basal medium^34–36^. However, only a few studies have investigated the changes in morphological structure and endogenous hormones that occur during somatic embryogenesis.

Despite its perennial nature, tea plants still confronted difficulties in obtaining intact plants during their tissue culture process, which affected tea plants propagation. In order to study the pathways or factors that played key roles in tea plants somatic embryogenesis. In this study, transcriptomics was used to study the changes in the functions of genes of metabolic pathways and their regulation during somatic embryogenesis in order to gain an understanding of the mechanism of somatic embryogenesis in tea, which will help to further elucidate the molecular mechanisms controlling somatic embryogenesis in tea and provide ideas for reproduction and genetic engineering of tea.

## Results

### Quality assessment of transcriptome sequencing for tea embryogenesis

61.353G of raw data were obtained from transcriptome sequencing of nine tea samples (Table S1). The raw data for all nine samples were more than 96% for Q20 and 91% for Q30. The raw reads GC content of the nine samples ranged from 45.115 to 47.855%. The clean data mapping rates to the “shuchazao” genome were 90.59–93.78%. Non-splice reads and splice ratio reads were 51.1–55.42% and 26.53–33.12%, respectively. Tea embryogenesis transcriptome sequencing data with high quality and mapping ratios were suited for RNA analysis. Analysis of the correlation of gene abundance between samples showed that the Spearman correlation coefficients between samples were all greater than 0.81 (Fig. S1).

### Differential gene expression in different tea embryogenesis stages

**Figure 1:**
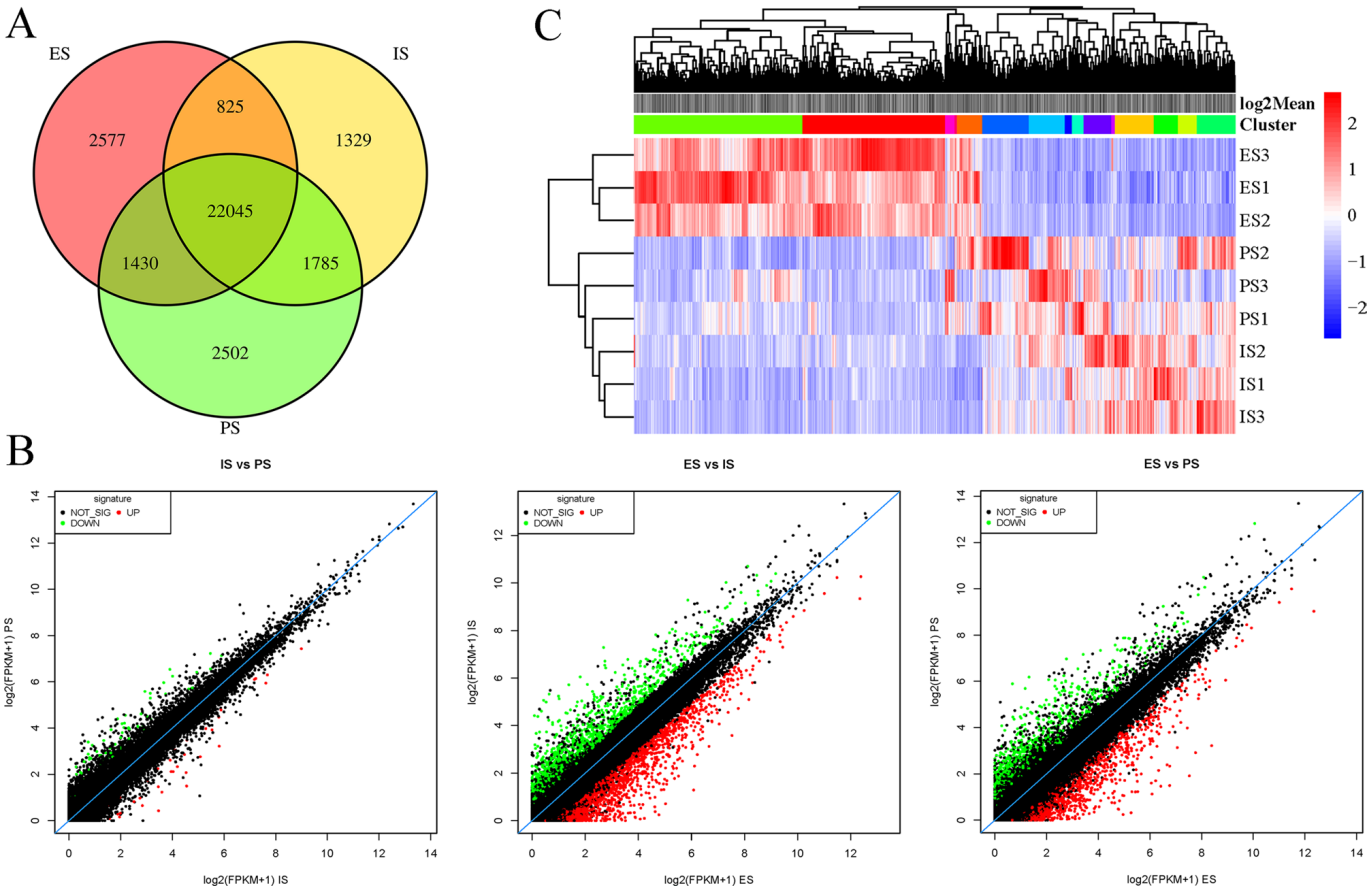
Correlation analyses of gene expression in the somatic embryogenesis stages of tea. (**A**) Venn diagram of gene expression at different stages of occurrence. (**B**) Scatter plot of DEGs in different stages. (**C**) The DEGs cluster heat map between different stages. *PS* preliminary stage, *IS* intermediate stage, *ES* embryoid stage.

27,765, 25,984, 26,877 genes were expressed in the stages of PS, IS and ES, respectively. 22,045 genes were co-expressed and 2502, 1329 and 2577 genes were uniquely expressed in PS, IS and ES, respectively (Fig. 1A). The numbers of DEGs for IS_PS, ES_IS, and ES_PS were 109, 2848, and 1697, respectively (Fig. 1B). IS_PS, ES_IS and ES_PS up-regulated DEGs were 32, 1721 and 960 and down-regulated DEGs were 77, 1127 and 737, respectively. The results of the cluster analysis showed that the differences in gene expression in ES_IS were significantly greater than those in IS_PS (Fig. 1C).

### Functional studies on DEGs during tea embryogenesis stages

**Figure 2:**
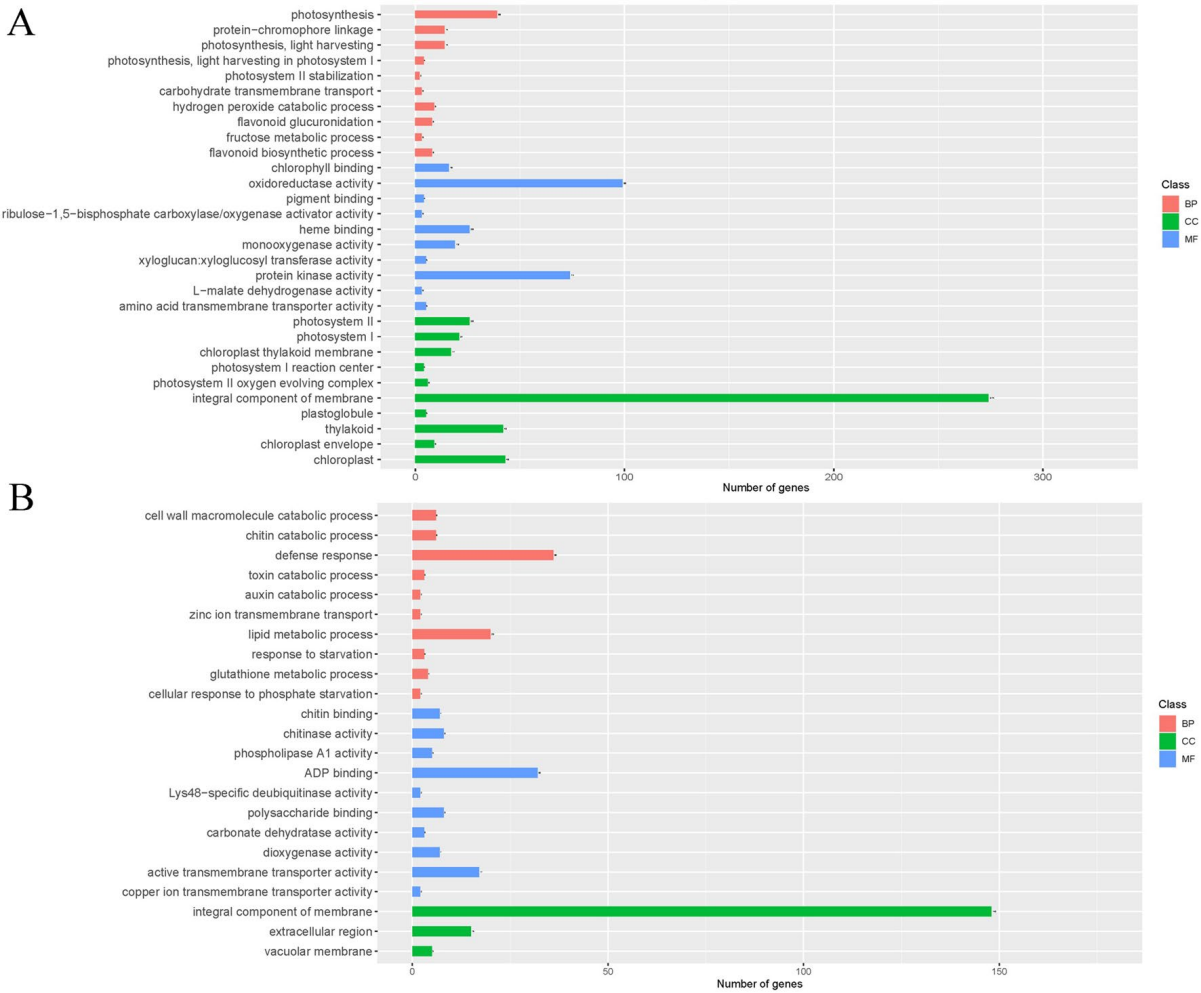
The function analysis of GO annotation for ES_PS stage. (**A**) Up-regulated genes enrichment analysis results. (**B**) Down-regulated genes enrichment analysis results.

GO annotation was used to study the DEGs function during tea embryogenesis (Fig. 2, Table S2). The main functions of the up-regulated DEGs in ES_PS were mainly enriched in photosynthesis, oxidoreductase activity, protein kinase activity, photosystem, integral component of membrane, thylakoid and chloroplast, and the number of related up-regulated genes were all greater than 39. The main functions of the down-regulated DEGs in ES_PS were mainly enriched in defense response, lipid metabolic process, ADP binding, active transmembrane transporter activity and integral component of membrane, and the number of related down-regulated genes were all greater than 17.

ES_IS was the main process of ES_PS differential, and ES_IS significantly up-regulated DEGs functions were similar to ES_PS but with increased function of carbohydrate metabolic process (Table S2). However, down-regulated DEGs functions had significant differences, and these functions were significantly up-regulated in IS_PS. The main functions of the down-regulated DEGs in ES_IS were mainly enriched in monooxygenase activity, heme binding, transferase activity, transferring hexosyl groups, iron ion binding, oxidoreductase activity, DNA binding, nucleus, intracellular membrane-bounded organelle, membrane-bounded organelle, oxidoreductase activity, acting on paired donors, with incorporation or reduction of molecular oxygen, and the number of related down-regulated genes were all greater than 28. IS_PS had a significant down-regulation of a large number of DEGs enrichment functions and a non-significant up-regulation. Molecular and cellular component functions in IS_PS were performed as down-regulation, including adenyl ribonucleotide binding, anion binding, protein kinase activity, cell periphery, membrane, integral component of membrane, intrinsic component of membrane.

### Pathway studies on DEGs during tea embryogenesis stages

**Figure 3:**
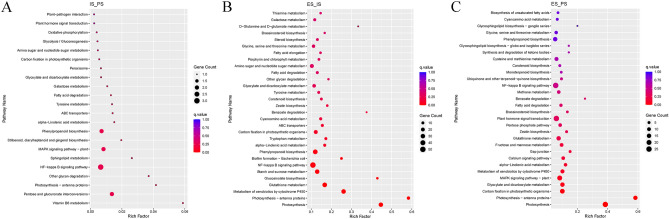
The enrichment analysis of KEGG pathway for the somatic embryogenesis stages of tea. (**A**) The pathway result for IP_PS stage. (**B**) The pathway result for ES_IS stage. (**C**) The pathway result for ES_PS stage.

KEGG database was used to analyze tea embryogenesis DEGs pathway annotation (Fig. 3). IS_PS DEGs were mainly enriched in Vitamin B6 metabolism, Pentose and glucuronate interconversions, and Photosynthesis—antenna proteins, but the enrichment abundance was much lower than that of ES_IS and ES_PS (Fig. 3A). ES_IS DEGs were mainly enriched in photosynthesis, photosynthesis-antenna proteins, metabolism of xenobiotics by cytochrome P450, glucosinolate biosynthesis and glutathione metabolism (Fig. 3B). The abundance of ES_PS DEGs enrichment pathways were similar to those of ES_IS, with the highest abundance being photosynthesis-antenna proteins and photosynthesis (Fig. 3C).

GSEA analysis was used to further identify the functional pathways for embryogenesis. Ribosome and SNARE interactions in vesicular transport pathways genes expression were all significantly upregulated during IS_PS. During ES_PS and ES_IS, photosynthesis and photosynthesis-antenna proteins pathways genes expression were significantly up-regulated. In addition, ribosome pathway genes were significantly up-regulated during ES_PS, steroid biosynthesis and NF-kappa B signaling pathway genes were significantly up-regulated during ES_IS.

**Figure 4:**
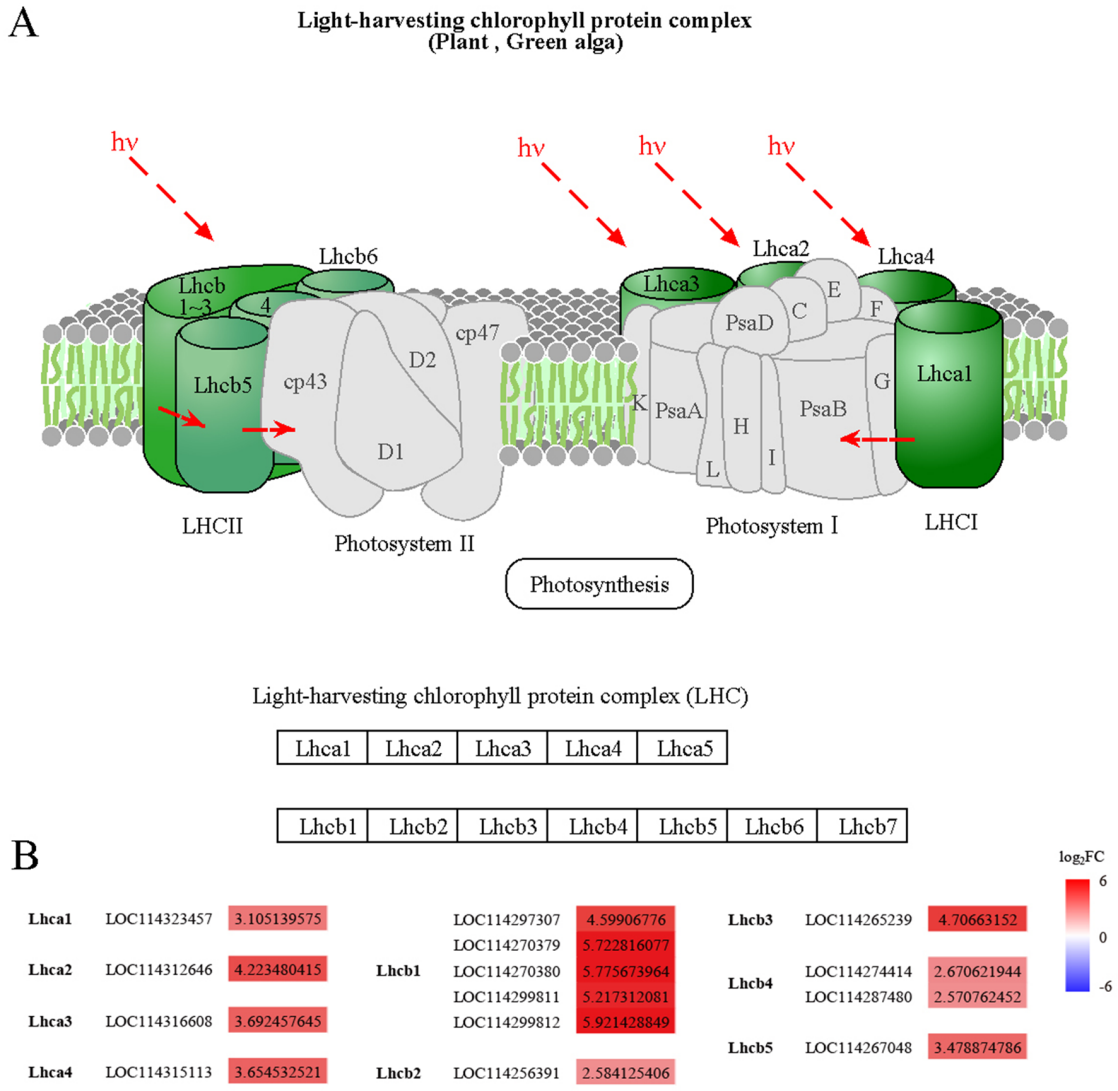
Gene expression regulates photosynthesis. (**A**) Light-harvesting chlorophyll protein complex model. (**B**) Expression of genes involved in light-harvesting chlorophyll protein complex.

There were 25 transcription factors in the Photosynthesis-antenna proteins pathway, 14 of which were significantly differentially expressed in the ES_PS process (Fig. 4). All 14 DEGs were significantly up-regulated and all log_2_FC were greater than 2.57. Nine of the 12 light-harvesting chlorophyll protein complexes were significantly up-regulated and three were not significantly differentially expressed during ES_PS. The five *Lhcb1* genes (*LOC114297307*, *LOC114270379*, *LOC114270380*, *LOC114299811*, *LOC114299812*) were significantly up-regulated during embryogenesis and all log_2_FC were greater than 4.59.

## Discussion

*Camellia sinensis* is an essential cash crop^37^, but researches into the breeding and propagation of superior tea varieties has been relatively slow due to the limitations of its own characteristics^38^. Somatic embryogenesis is a key factor in plant regeneration, and tea somatic embryogenesis occurs in both direct and indirect ways. Indirect occurrence usually takes cotyledons, shoot tips or petals as explants to induce somatic embryos. The tea somatic embryos were classified into three states according to the morphology of the cotyledon explants: the preliminary stage, the intermediate transition and the somatic embryo state. Transcriptomics techniques have been widely used in the field of plants and have played a crucial role in plant gymnosperm embryogenesis^39–42^. In this study, transcriptomics was used to investigate the mechanisms of gene expression regulation during somatic embryogenesis in tea plants.

As tea plant somatic embryogenesis was autotoxic, resulting in its inability to produce regenerating plants, key segments of the embryogenesis process were explored in stages. Transcriptome analysis showed that the numbers of DEGs in ES_IS stage were much larger than that in IS_PS and ES_PS, and the up-regulated DEGs in ES_IS stage was much larger than the down-regulated DEGs. Genes related to growth, development and metabolism were mostly found in the up-regulated DEGs. At the same time, the results of cluster thermogram also showed that ES_IS stage played a key role in the process of tea somatic embryogenesis compared with IS_PS. In addition, unlike in tea plants, the vast majority of differentially expressed genes in maize somatic embryogenesis were clustered in the immature embryo to the embryogenesis healing tissue stage, with a tendency for up-regulation of expression during the dedifferentiation process^14^.

Based on GO enrichment analysis, DEGs upregulated at the ES_PS stage were significantly enriched in cellular components such as overall membrane composition, molecularly functional protein kinase activity and photosynthesis in biological processes. For example, SNF1/AMPK-associated protein kinases were linked to downstream gene expression, physiology and development through signalling^43^; Mitogen-activated protein kinases (MAPKs) were organised into complex networks for signalling and thus regulating plant growth and development^44^. In contrast, the ES_IS stage, as the main process of embryogenesis in tea somatic cells, had similar functions to other up-regulated DEGs, except for the carbohydrate metabolic process. Carbohydrates serve as the main component of cellular structure and could provide the main energy for organism development^45^, as well as modifying lipids and proteins, altering their structure and functions. This might also account for the significant differences between IS_PS and ES_IS. The DEGs that were down-regulated during ES_IS were significantly different from those that occur throughout and, interestingly, these functions were significantly up-regulated in IS_PS. Light capture in cellular component-enriched functions such as photosystem I and biological processes photosystem I could promote carbohydrate biosynthesis^46^ and might provide the material basis for carbohydrate metabolic processes in ES_IS. The pathway including carbohydrate metabolism was detected to maintain embryogenesis potential in *Larix kaempferi* (Lamb.) Carr^47^. Carbohydrate and metabolic pathways were recognised as representative overexpression pathways in early embryogenesis of maritime pine and were valuable resources to further support the improvement of trophic reproduction with this specie^48^. Similar to tea plants, crucial amino acid biosynthetic pathways were identified in conifer embryonic development^49^, suggesting that metabolism might be indispensable in somatic embryo formation. Furthermore, in cotton somatic embryogenesis, genes were significantly enriched in metabolic pathways and secondary metabolite biosynthesis^50^. Genes involved in somatic embryogenesis in different species regulated embryonic development by enriching in different pathways.

Pathway enrichment analysis of DEGs showed that far fewer pathways were enriched during IS_PS than ES_IS or ES_PS, which also indicated that ES_IS was a critical stage in tea somatic embryogenesis. ES_IS and ES_PS shared similar levels of enrichment, with photosynthesis and photosynthetic antennae proteins being the most enriched. Photosynthesis and photosynthetic antenna protein pathway gene expression were also significantly up-regulated in the GSEA analysis of ES_IS and ES_PS, indicating that photosynthesis and its antenna protein pathway had an important role in tea somatic embryogenesis. Antenna proteins performed a key regulatory role in light capture in photosynthesis^51^ and had the role in protecting plants from high light damage and regulating the capture of solar energy by plants and its transfer to reaction centres^52,53^. Light contributed to the early development of the somatic cell embryo, and the significant enrichment of photosynthesis in ginkgo somatic cell embryo formation promoted the induction of the cotyledonary somatic cell embryo^54^. Knockdown of five genes of *Lhcb1* in *Arabidopsis* resulted in chlorophyll loss and delayed growth^55^. Down-regulation of *Lhcb1*, *Lhcb3* and *Lhcb5* genes expression levels in cucumber affected its photosynthesis^56^. In contrast, significant up-regulation of the five *Lhcb1* genes during embryogenesis might contribute to their light capture and photosynthesis. To date, few genes involved in embryogenesis have been characterised in tea plants, and their expression was crucial to acquisition in embryogenic competence and expression in somatic embryogenesis. In some cases, the expression of these genes might determine somatic cell fate changes. Therefore, for the discovered *Lhcb1* gene could be considered as a biomarker for somatic embryogenesis in tea plants, which could be validated for biological function and then clonally characterised to advance tea breeding and propagation strategies. Transcription factors specifically bind to cis-elements of target gene promotered to regulate gene transcription^57^, and all transcription factors in the photosynthetic antennae protein pathway were significantly up-regulated during ES_PS. The mechanisms of transcriptional regulation of these transcription factors in tea somatic embryogenesis need to be further revealed. Photosynthesis actes as an irreplaceable role throughout the plant life cycle^58^, providing continuous energy income for metabolism^59^ and growth and development. The significant enrichment of the photosynthetic system pointed to an essential role for its regulation in somatic embryogenesis. It was worthwhile to further investigate how these significantly related differential genes and enriched pathways regulated tea somatic cell embryogenesis, and the transcriptional regulatory mechanisms of the transcription factors need to be further unravelled. Further elucidation regarding the precise roles of these pathways will facilitate the molecular understanding of tea somatic cell embryogenesis and the development of reproductive strategies. In addition, future studies should not be limited to a single omics, proteomics that identified specific proteins associated with somatic embryogenesis and development, and epigenetics techniques to serve the researches.

## Materials and methods

### Sample collection

The three occurrence stages used in this study to form tea somatic embryos were provided by Shandong Agricultural University. *Camellia sinensis* cv. Jinxuan tea seeds were used as the primary material for the somatic embryogenesis process, where cotyledon cuts were induced into the somatic embryo state, and all materials used in the experiments were taken from the same tea seeds. Part of the cotyledons were induced directly into the somatic embryo, a portion of the cotyledons were in the elevated state, and a percentage of the cotyledons remained in the initial state during the 6 months of induction. The outermost layer of the cotyledons was cut for the PS, the IS was cut for the uplifted portion of the leaf, and the ES was selected for the somatic embryo that had already been induced. Materials from all three states of the same tea seed were sampled with a scalpel and immediately fixed in liquid nitrogen and stored at – 80 °C^60^. Transcriptome sequencing was performed on three groups of material and three replicates were used to undertake sequencing.

### Transcriptome sequencing and assembly

RNA with Poly-A structure in eukaryotic total RNA was enriched using the TIANSeq mRNA capture kit (TIANGEN Biotech). Nanodrop 2000 spectrophotometer (Thermo Fisher Scientific) was used to determine the concentration of RNA samples and assess their purity^61^. Agilent 2100 Bioanalyser and 2100 RNA nano 6000 Assay Kit (Agilent Technologies) were used to assess the integrity of the RNA samples^62^. Using the captured RNA as the starting sample, TIANSeq Fast RNA Library Kit (Illumina) was used to construct the transcriptome sequencing libraries. The transcriptome sequencing library was constructed through RNA randomly fragmentation, cDNA strand 1/strand 2 synthesis, end repair, A-tailing, ligation of sequencing adapters, size selection and library PCR enrichment. Library concentration was first quantified using Qubit 2.0 fluorometer (Life Technologies), and then diluted to 1 ng/µl before checking insert size on an Agilent 2100 and quantifying to greater accuracy by quantitative PCR (Q-PCR) (library activity > 2 nM). The clustering of the index-coded samples was performed on a cBot Cluster Generation System using TruSeq PE Cluster Kit v3-cBot-HS (Illumina) according to the manufacturer’s instructions. After cluster generation, the library preparations were sequenced on an Illumina sequencing platform and 150 bp paired-end reads were generated.

### Identification and analysis of differentially expressed genes

The raw data in fastq format (raw reads) were first processed by an internal perl script. In this step, clean data (clean reads) were obtained by removing reads containing articulators and trimming low quality bases with Trimmomatic^63^. Quality control was performed using FASTQC^64^. Clean data were also calculated for Q20, Q30 and GC content. All downstream analyses were based on high quality clean data. Hisat2 v2.0.5^65^ was used to construct the “Shuchazao” reference genome^66^ (http://tpia.teaplant.org/download.html, accessed 5 April 2023) indexed on 5 April 2023. The paired-end clean reads were aligned with the reference genome and the mapping information was calculated.

Differential expression analysis of tea somatic embryo samples at each stage was performed using the DESeq2 R package (1.16.1)^67^. Genes identified by DESeq2 with a P value < 0.05 were classified as differentially expressed genes. DEGs were analyzed separately by Gene Ontology (GO) (http://www.Gene Ontology.org, accessed 5 April 2023) and the Kyoto Encyclopedia of Genes and Genomes (KEGG) database (www.kegg.jp/kegg/pathway.html, accessed 5 April 2023) were annotated and enriched to obtain functional and pathway results for DEGs^68,69^. ClusterProfiler^70^ and ggplot2^71^ R packages were used for GO and KEGG enrichment analysis and visualization.

### Supplementary Information

Supplementary Figure S1.

Supplementary Table S1.

Supplementary Table S2.

## Data Availability

Data from the current study are available from the corresponding author upon reasonable request.
