# Supplementary figures and images for: Transcriptome analysis revealed enrichment pathways and regulation of gene expression associated with somatic embryogenesis in Camellia sinensis
Source: Sci Rep. 2023 Sep 24;13:15946. doi: 10.1038/s41598-023-43355-9 (PMC10518320; doi:10.1038/s41598-023-43355-9)

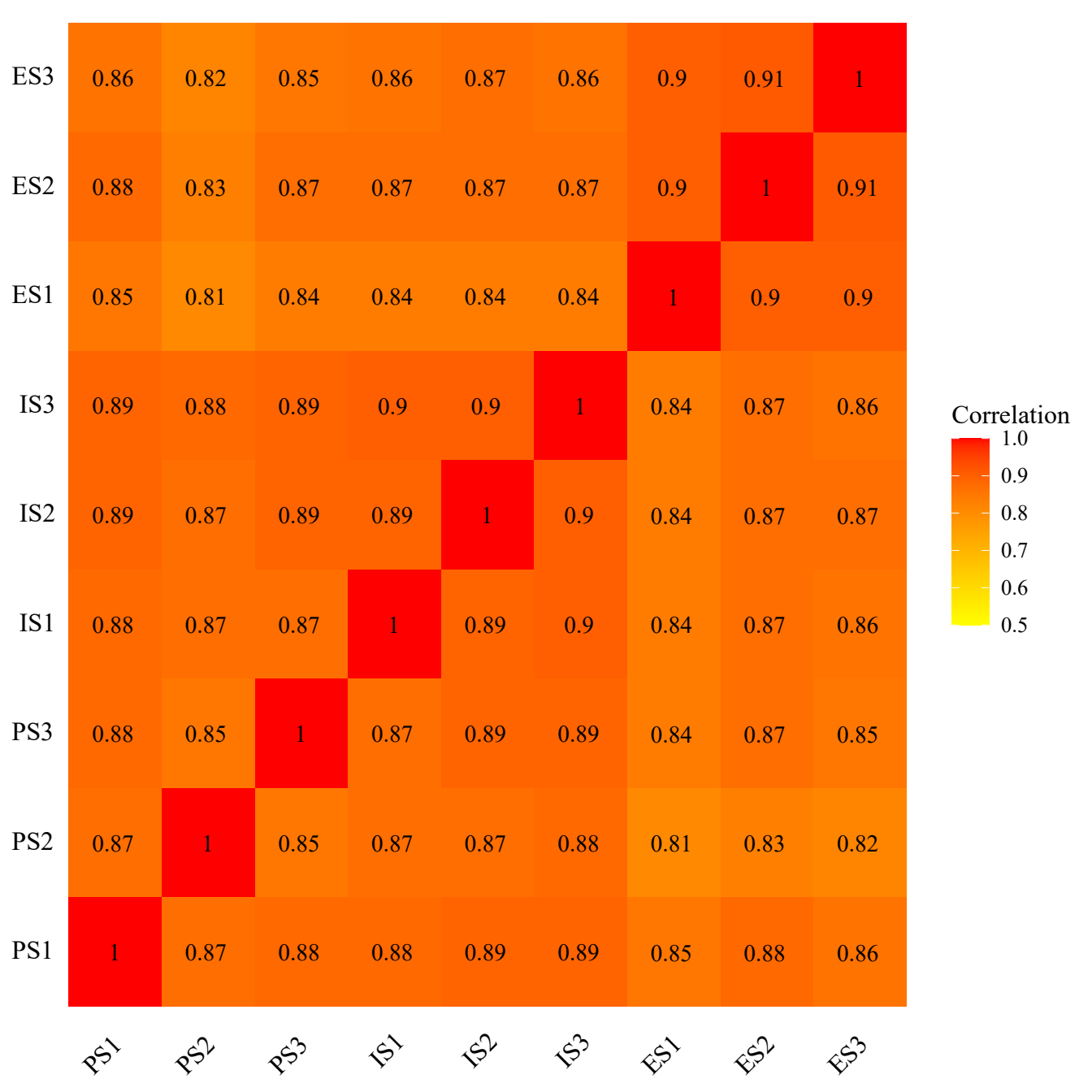

Supplement: Supplementary file 1 — Supplementary Figure S1. [file 41598_2023_43355_MOESM1_ESM.pdf]
